# Supplementary material for: Mechanisms governing the pioneering and redistribution capabilities of the non-classical pioneer PU.1
Source: Nat Commun. 2020 Jan 21;11:402. doi: 10.1038/s41467-019-13960-2 (PMC6972792; doi:10.1038/s41467-019-13960-2)
Supplement: Supplementary file 7 — Source data [file 41467_2019_13960_MOESM7_ESM.zip › Source_Data/FigureS3/FigureS3H_MotifScanOutput/homerResults/motif34.similar.html]

motif34

## Information for motif34

A
G
T
C
A
C
T
G
A
C
G
T
C
T
A
G
A
C
T
G
A
C
T
G
G
T
C
A
  
Reverse Opposite:  

C
A
G
T
T
G
A
C
A
G
T
C
A
G
T
C
C
G
T
A
A
G
T
C
C
T
A
G
  

|  |  |
| --- | --- |
| p-value: | 1e-24 |
| log p-value: | -5.606e+01 |
| Information Content per bp: | 1.890 |
| Number of Target Sequences with motif | 1597.0 |
| Percentage of Target Sequences with motif | 41.31% |
| Number of Background Sequences with motif | 14799.7 |
| Percentage of Background Sequences with motif | 33.04% |
| Average Position of motif in Targets | 341.2 +/- 339.1bp |
| Average Position of motif in Background | 209.1 +/- 139.9bp |
| Strand Bias (log2 ratio + to - strand density) | 0.0 |
| Multiplicity (# of sites on avg that occur together) | 1.43 |
| Motif File: | file (matrix) reverse opposite |

### Similar de novo motifs found

|  |  |  |  |  |  |  |  |
| --- | --- | --- | --- | --- | --- | --- | --- |
| Rank | Match Score | Redundant Motif | P-value | log P-value | % of Targets | % of Background | Motif file |
| 1 | 0.898 | G T A C A C T G C A G T C A T G C T A G T C A G G C T A C T G A T G C A | 1e-17 | -40.314596 | 2.46% | 0.81% | motif file (matrix) |
| 2 | 0.792 | A G T C A C T G A C T G A C T G A C T G A C T G G T C A G C T A | 1e-15 | -35.988937 | 5.51% | 2.91% | motif file (matrix) |
